# Acute Effects of Ketamine Infusion on Postoperative Mood Scores in Patients Undergoing Dilation and Curettage: A Randomized Double-Blind Controlled Study

**DOI:** 10.1155/2021/6674658

**Published:** 2021-03-23

**Authors:** Raham Hasan Mostafa, Ahmed Mohamed Khamis, Ashraf Nabil Saleh, Yehia Mamdouh Hassan Mekki, Mohamed Mohamed Kamal, Ismail Mohammed Ibrahim, Mohamed Abdulmohsen Abdulnaiem Ismaiel

**Affiliations:** Department of Anesthesia, Intensive Care and Pain Management, Ain Shams University, Cairo, Egypt

## Abstract

**Background:**

Emotional and psychological effects following abortion are more common than physical side effects and can range from mild regret to more serious complications such as depression. In the last decade, it has been suggested that low dose of ketamine is a fast-acting antidepressant.

**Purpose:**

The aim of this study was to investigate the impact of intraoperative ketamine infusion on postoperative mood score in patients undergoing Dilation and Curettage (D&C) under spinal anesthesia. We hypothesized that a single low-dose administration of ketamine infusion during D&C surgery can improve mood scores in the immediate postoperative period.

**Methods:**

A prospective, randomized, double-blind, parallel-group, placebo-controlled trial. The study included a total of 60 patients, ≥18 years, physical status ASA II, with up to 12-week gestation undergoing elective D&C surgery. Patients were divided randomly into a ketamine group (group K) and a control group (group C). In group K, 0.4 mg/kg ketamine was given as a continuous infusion over 20 min intraoperatively. *Main Outcome Measure*. Profile of Mood States (POMS) was recorded preoperatively and 2 hours postoperatively.

**Results:**

There were no differences in preoperative POMS between the two groups. Mean postoperative POMS of group K was lower than that of group C indicating mood improvement. Ketamine group patients showed higher sedation score and increased, although self-limiting, psychedelic phenomena than the control group.

**Conclusion:**

Observed data here support an acute effect of ketamine on mood but any further claim will be speculative. Further future studies exploring postoperative mood scores after 24 hours post-infusion are needed. This trial is registered with PACTR201907779292947.

## 1. Introduction

Mood is believed to have a direct impact on mental and physical health. Mood has been believed to be a multidimensional construct including not only depressive symptoms, but also anger, sadness, fear, tension, joy, and guilt. Research published in 2011 [1] concluded that there was a “moderate to highly increased risk of mental health problems after abortion.” The researchers suggested that undergoing a termination increased the risk of mental health problems by 81% that may be partly due to hormonal changes. They also attributed 10% of this risk to termination itself [1]. Mood is commonly measured using the Profile of Mood States (POMS) which is a psychological rating scale published in 1971 used to assess transient, distinct mood states [2].

Since the discovery of ketamine by Domino et al. [3], research had been done to determine its various clinical uses [4]. Recent studies suggest that a single low-dose administration of ketamine can provide, within a few minutes of its initiation, a long-lasting effect on mood [5]. Ketamine has emerged as a rapid powerful antidepressant with enduring effects that last a week after a single subanesthetic dose in the range of 0.3 to 0.5 mg/kg. In low doses, it relieves depression, but in higher doses, it can cause patients to feel an uncomfortable sense of dissociation from the body similar to a near death experience [6, 7]. Interestingly, the half-life of ketamine is only 3 hours, suggesting that its antidepressant effect is unrelated to continuous blocking of N-methyl-d-aspartate (NMDA) receptors [8]. Induction of transitory psychotic episodes is the main drawback of ketamine [9].

Given the aforementioned data, we hypothesized that intraoperative subanesthetic ketamine infusion in a vulnerable patient group would have favorable effects on postoperative mood scores after Dilation and Curettage (D&C) procedure.

The primary endpoint was the response rate at 2nd postoperative hour, and the secondary endpoints included the incidence of postoperative side effects. We chose regional anesthesia and not general anesthesia (GA) for 2 reasons: first, the effect of ketamine differs from awake to anesthetized [10]; second, to eliminate effect of propofol and isoflurane which have antidepressant effects and might affect mood scale [11, 12].

## 2. Materials and Methods

### 2.1. Ethics

Ethical approval for this study (FMASU R 37/2019) was provided by the Ethics committee of Ain Shams University Hospital, Abbasia, Cairo, Egypt (Chairperson Prof F. Tash) on 10/7/2019. The study was prospectively registered with Pan African Clinical Trial Registry (PACTR) with Registration Number PACTR201907779292947 on 16/7/2019 in accordance with WHO and ICMJE standards. Written informed consent was obtained from all subjects.

### 2.2. Study Population

Sixty patients, 18 years or older, physical status ASA II, with up to 12-week gestation, who were undergoing D&C procedure for incomplete or retained abortion, had participated in this randomized, double-blind study.

#### 2.2.1. Exclusion Criteria

1. Usage of sedative drugs or substance abuse.
2. Patients who had comorbid psychiatric disorders such as depression or anxiety disorders.

### 2.3. Patients' Recruitment and Randomization

Patients were recruited after admission to the hospital, the same day they scheduled for surgery. Randomization was performed using computer-generated random number tables in opaque sealed envelopes prepared by an anesthesiologist who was not part of the study. The randomization was performed on 1 : 1 ratio according to a computer-generated table. Group assignments were enclosed in sealed, opaque, sequentially numbered envelopes by a research nurse not involved in the study. All the attending anesthesiologists, the patients, and the data collectors were blinded to group assignment throughout the entire study period. On the scheduled time of operation, the head nurse (who was not part of the study) opened each envelope just before surgery, prepared the infusion solution, and handed it to the anesthesiologist. He was then determined for collecting perioperative data.

The patients were randomized to 2 groups: C (control group) and K (ketamine group).

Group C: patients received 50 ml 0.9% saline over a period of 20 minutes.

Group K: patients received 0.4 mg/kg ketamine diluted in 50 ml 0.9% saline over a period of 20 minutes.

Ketamine infusion dose was given over 20-minute duration, whether the surgical procedure had ended or not. This dosing regimen is in accordance with existing guidelines [13].

### 2.4. Anesthesia and D&C Procedure

On arrival to the operating room, all patients received standard continuous monitoring. All patients were premedicated with intravenous (IV) midazolam (0.03 mg/kg) and 1 mg IV granisetron before starting the procedure. In both groups, all patients were given spinal anesthesia according to standard practice. Patients were then positioned in the lithotomy position after a satisfactory spinal block was verified and the study infusion was started for 20 minutes. Infusions would be stopped if any uncontrollable complication occurred. Patients were asked to assign their POMS preoperatively and 2 h postoperatively.

### 2.5. The Profile of Mood States (POMS)

This scale was developed by McNair et al. [14]. The POMS allows both positive and negative states to be measured and observed in a clinical setting. This enables health professionals to identify the patient's current clinical condition [15]. Furthermore, it may be used to measure the effectiveness of treatments. The first edition of the Profile of Mood States scale is known as the POMS-Standard version or the POMS-long form [14]. The brevity needed in some research environments (where there is a limited amount of time available for data collection) has spawned many shortened versions of the POMS. Of the shortened versions, the Profile of Mood States-Adolescents [16] has been subjected to perhaps the most meticulous validation process. The adolescent measure is a simpler and shorter version of the adult measure [16]. In 2003, Terry and his colleagues had constructed the validity of the POMS-A to adults and adult athletes age groups. It has been shown to be satisfactory [17].

POMS can be administered and measured through written or online forums. The POMS usually takes between 5 and 7 minutes to complete. The POMS measures six different dimensions of mood. Score for each item is recorded as 0 for “not at all” up to 4 for “extremely,” except for the two esteem-related affect subscales which are reverse-scored. A Total POMS score is calculated by summing the totals for the negative subscales (tension, depression, fatigue, confusion, anger) and then subtracting the totals for the positive subscale (vigor and esteem-related affect) [17]. Lower scores indicate more stable mood profiles.

### 2.6. Outcome Measures

Our primary outcome was mood changes assessed by POMS, preoperatively and 2 h postoperatively.

Secondary outcomes were as follows:

1. Mean blood pressure (MBP) and heart rate (HR) that were measured preoperatively and then every 5 minutes till end of the operation. Postoperatively, they were recorded every 60 minutes for 2 hours in PACU.
2. The duration of surgery in minutes.
3. Presence of psychedelic phenomenon [18] intraoperatively (during infusion) and/or postoperatively.
4. It is a temporary altered state of consciousness that might be induced by ketamine infusion. The subjective phenomenon may be in the form of perceptual disorders, a sense of detachment from their bodies (dissociation), and a sense of relaxation or well-being.
5. Perioperative headache and diplopia.
6. The patient satisfaction was assessed postoperatively at the 2^nd^ hour. Patient satisfaction was rated on a scale of 1 to 4 (1 = perfect, 2 = good, 3 = moderate, 4 = bad).
7. Assessment of perioperative sedation level using University of Michigan Sedation Scale (UMSS) (Table 1) [19]. The UMSS is a simple observational tool that assesses the level of alertness on a five-point scale ranging from zero (wide awake) to 5 (unarousable with deep stimulation).
8. It was assessed for 2 hours, every 60 minutes.

### 2.7. Statistical Analysis

Using G power program, setting alpha error at 5% and power at 80%, assuming an effect size of 0.8 (Cohen's d) between the two studied groups (ketamine and control) as regards the mean change in POMS after D&C procedure produced a sample size of 30 cases per group, taking into account 20% dropout rate.

The collected data were coded, tabulated, and statistically analyzed using IBM SPSS statistics (Statistical Package for Social Sciences) software version 22.0, IBM Corp., Chicago, USA, 2013. Descriptive statistics were done for quantitative data as mean ± SD (standard deviation) for quantitative normally distributed data, while it was done for qualitative data as number and percentage. Inferential analyses were done for quantitative variables using Shapiro–Wilk test for normality testing, independent *t*-test in cases of two independent groups, and repeated measures analysis of variance (RMANOVA) for repeated measures within independent groups and Chi square test was done for differences between proportions and Fisher's Exact test in cases of small expected cell counts. The level of significance was taken at *P* value < 0.050; otherwise it was non-significant.

## 3. Results and Discussion

### 3.1. Results

**Figure 1:**
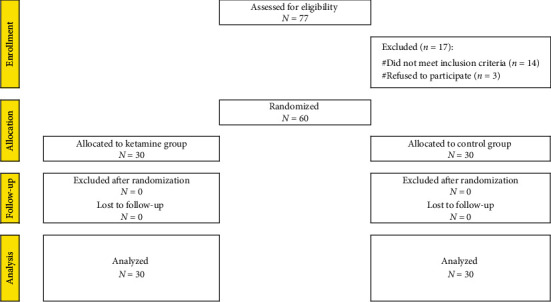
Flow chart of the studied cases.

The current prospective, placebo-controlled, randomized, double-blind trial was conducted at Ain Shams University Hospital, Egypt, from July 2019 to August 2020. This trial followed the CONSORT statement. A total of 77 patients were assessed for eligibility. 14 patients did not meet the inclusion criteria and 3 patients refused to participate (Figure 1).

**Figure 2:**
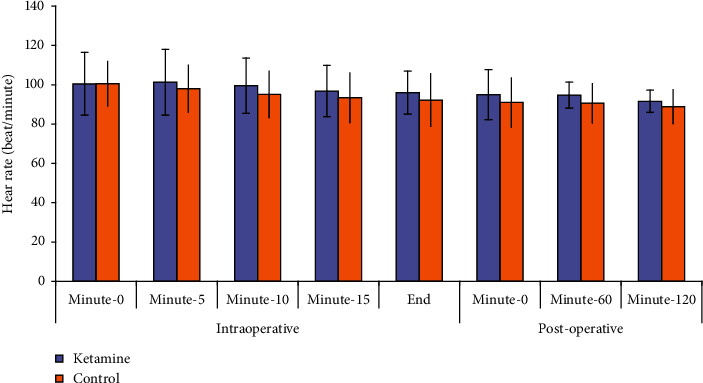
Change in mean heart rate (HR) in both study groups.

**Figure 3:**
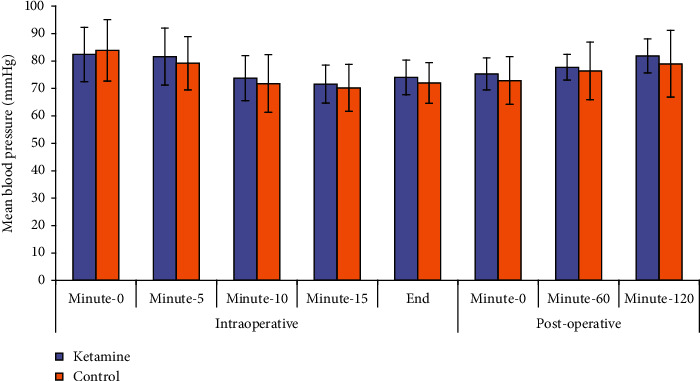
Change in mean blood pressure (MBP) in both study groups.

Perioperative characteristics of patients are shown in Table 2. There were no significant differences between the studied groups regarding age, weight, body mass index (BMI), gravidity, and American Society of Anesthesiologist-Physical Status (ASA-PS) and surgery duration. All patients completed their high educational level. As shown in Figures 2 and 3, heart rate and mean blood pressure were non-significantly higher in ketamine group, but both of them significantly decreased by study time points without significant interaction between study groups and time.

Tables 3 and 4 show that, after 2 hours post-infusion, there were significant differences between groups in all total subscales (except total anger subscale), and significant differences within subjects in all total subscales (except both of total tension and total confusion subscale). Finally, there was significant interaction between group and time in all total subscales. Each subscale's underlying items' statistical variability can be viewed in Tables 3 and 4.

**Figure 4:**
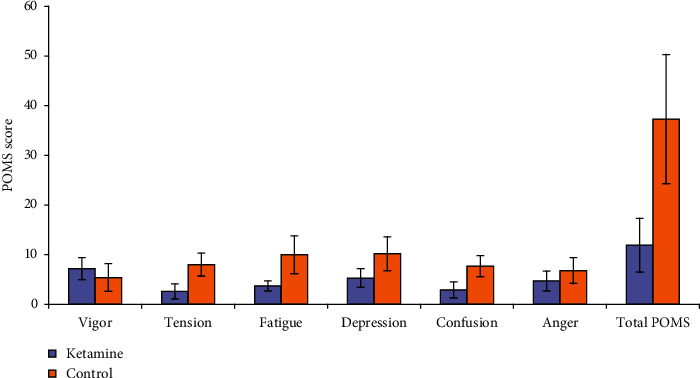
Comparison according to POMS subscales in both groups.

Regarding Total POMS score, there was significant difference between groups and within subjects, as well as significant interaction between group and time. As noticed, total POMS score was significantly lower in ketamine group than control group, indicating improvement in mood (Tables 3 and 4, Figure 4)

Table 5 shows that postoperative sedation was higher in ketamine group; the differences were significant except at minute 120. Satisfaction score was significantly lower (better) in ketamine group. Intraoperative and postoperative psychedelic phenomena, headache, and diplopia were more frequent in ketamine group.

### 3.2. Discussion

Approximately 20% of patients experience a depressed mood during the perioperative period, affecting their recovery and prognosis. The negative impact of this has received insufficient attention from clinicians [20]. In this study, we investigated the effect of a single, low-dose ketamine given as infusion over 20 minutes, on mood after surgery (assessed by POMS). We found that ketamine had lowered postoperative total POMS score significantly which could be an indication of mood improvement.

The mechanism of ketamine's effect on mood is unclear, but there is evidence demonstrating that ketamine can reverse synaptic dysfunction induced by stress. This raises the probability that intraoperative administration of ketamine might improve postoperative mood and enhance psychological strength in the setting of surgical stress [10]. Autry and Monteggia [21] suggested that the rapid rise of brain derived neurotrophic factor (BDNF) was one of the important mechanisms of ketamine's antidepressive effect.

Very few studies investigated the impact of ketamine's subanesthetic dose on mood in non-depressed patients. Notably, their results were inconsistent.

An earlier study done by Långsjö and their colleagues [22] in 2003 in Finland explored the effects of subanesthetic doses of ketamine on regional CBF (rCBF), regional metabolic rate of oxygen (rCMRO2), and regional cerebral blood volume (rCBV) on 9 healthy male volunteers during three escalating concentrations of ketamine. At the end of each concentration level, volunteers' subjective feelings were rated using a modified Profile of Mood States scale. To be noted, Långsjö and their colleagues used a different POMS version than our study. In general, they found that higher ketamine dose was associated with more pleasantness and less anxiety score, while lower doses were associated with less anxiety and less vigilance scores. There was no change in anger, mood, cheerfulness, and depression subscales.

In another study done by Wang and their colleagues [13], they hypothesized that 0.4 mg/kg of ketamine over 20 minutes after laparoscopic bariatric surgery can improve pain and mood scores in the immediate postoperative period. They performed their study on 100 morbidly obese patients on which they assessed their mood by Beck Depression Inventory (BDI) and Montgomery–Asberg Depression Rating Scale (MADRS). In their study, they did not observe any difference in MADRS and BDI which could be attributed to MADRS and BDI usually being used for patients who suffer from serious depression so; they may not be suitable for the analysis of subtle depressive symptoms. On the other hand, they found patients in ketamine group experienced significant improvement in their comprehensive evaluation of pain, particularly the affective component of pain (anxiety, fear, unpleasant sensation), on postoperative day 2 (POD 2) using short-form McGill's Pain Questionnaire (SF-MPQ). These results also suggest that ketamine may be able to improve postoperative mood.

Another study done by Dalsasso and their colleagues [23] tested whether the combination of intravenous low-dose ketamine (0.4 mg/kg) to midazolam, clonidine, and ketorolac could provide adequate anesthesia in opioid free day case surgery in 500 medically free patients. The Profile of Mood State was used to assess mood. In their study, mood score decreased postoperatively (indicating improvement in mood) but with patients complaining most frequently of tiredness.

On the other hand, Xu and their colleagues [24] concluded that intraoperative low-dose ketamine (0.25 mg/kg) does not have a preventive effect from postpartum depression, in 330 parturients who undergone cesarean section under spinal anesthesia. Ketamine was given within 5 minutes following clamping of the neonatal umbilical cord. The primary outcome was the degree of depression, evaluated using the Edinburgh Postnatal Depression Scale (EPDS). The non-preventive effect of the single bolus dose of ketamine on postpartum depression may be due to the relatively small dose. In addition, even if a large dose of ketamine (i.e., greater than 0.25 mg/kg) can prevent postpartum depression, the potential neurotoxic effects on the newborn and the psychological side effects for the mother are still obstacles to its wide use. Finally, EPDS may not be sensitive enough and thus may need to be combined with other scales to enhance its accuracy in assessing postpartum depression.

Surprisingly, Nugent and their colleagues [25] found that ketamine had induced depressive symptoms in healthy controls. A total of 60 patients were enrolled in their study (35 unmedicated treatment-resistant subjects with major depressive disorder (MDD) and 25 healthy controls). Ketamine (0.5 mg/kg) and placebo infusions were administered two weeks apart in all patients. The Montgomery–Åsberg Depression Rating Scale (MADRS) was the primary outcome measure, assessing antidepressant effects of examined intervention. In subjects with major depressive disorder, ketamine showed robust, rapid, and relatively sustained significant improvements in depressive symptoms, while in healthy control subjects, they exhibited an unexpected mood-lowering effect and an increase in depressive symptoms. The increase in depressive symptoms was significant at 40, 80, and 120 minutes after infusion and at day 1 (primarily in the domains of anxiety and anhedonia).

In a unique study done by Micallef and their colleagues [26], they showed that a low dose of ketamine (0.5 mg/kg over 60 minutes) induced schizophrenic-like symptoms in healthy volunteers. This study was done on 12 normal subjects with 2 sessions separated by one week of wash-out to determine ketamine-induced effects on behavioral and emotional responses. Six different scales were assessed under a time frame of 180 minutes since the start of infusion; POMS was one of them. Specifically, regarding POMS, ketamine infusion had led to significant decrease in vigor subscale and a significant increase in both fatigue and confusion subscales after 15 minutes from the start of infusion. We have no explanation to this difference between our results and their results. But, this difference might be attributed to different time of assessment of POMS (15 minutes after the start of infusion), even before completion of the planned dose and time.

Intraoperative low-dose ketamine administration under general anesthesia showed also contradictory results [10, 20] and needs further research. Neurobiologically, it should be appreciated that the administration of subanesthetic ketamine during general anesthesia represents a radically different neurochemical milieu compared with the waking state [10].

Regarding ketamine's side effects, the first systematic review of the safety of ketamine in the treatment of depression after single and repeated doses has been published recently [27]. In general, the mentioned side effects (as psychiatric, psychotomimetic, cardiovascular, and neurological side-effect) resolve within 2 h after ketamine administration [28]. The incidence of adverse events was reported to be very low and mild with low-dose ketamine [22, 29]. In our study, we reported most of these side effects, in addition to sedation, all of which were self-limiting requiring minimal interference in the form of verbal reassurance and midazolam increments and this goes with other studies [13, 24]. In Dalsasso and their colleagues' study [23], they reported that clonidine and midazolam administration during anesthesia appeared to minimize the cardiovascular and psychotomimetic effects of ketamine.

In our study, heart rate and mean blood pressure were non-significantly higher in ketamine group. Ketamine is known to transiently increase hemodynamic parameters but usually no medication treatment is required. In a study done by Riva-Posse and their colleagues [30], blood pressure changes (observed when ketamine is administered over 40 minutes at 0.5 mg/kg for the treatment of depression) were small, well tolerated, and clinically insignificant. Considering the potential risks of ketamine, it is recommended that clinicians delivering the treatment be prepared to manage potential cardiovascular events should they occur [31].

In summary, no drug is without side effects. In our experience, the side effects of ketamine have been well tolerated and self-limited and resolve shortly. If used in the context of a protocol that sets clear rules and includes training of staff members, it can be very effective and safe.

## 4. Limitation

Observed data here support an acute effect of ketamine on mood but any further claim will be speculative. After analysis of observed data, we found that our choice of the 2-hour time point for the primary outcome was a major limitation of our study. As a result, we cannot extrapolate improved postoperative mental health without further future studies documenting postoperative mood scores (POMS) after 24 hours post-infusion; in particular, we noted that ketamine does have significant adverse effects, so that an improvement in mood that lasts for only several hours is likely not worth the adverse effects.

## 5. Conclusion

Observed data here support an acute effect of ketamine on mood but any further claim will be speculative. Further future studies exploring postoperative mood scores after 24 hours post-infusion are needed.

## Figures and Tables

**Table 1:** University of Michigan Sedation Scale (UMSS) [19].

Score	Patient state
0	Awake/alert
1	Minimally sedated: tired/sleepy, appropriate response to verbal conversation and/or sounds
2	Moderately sedated: somnolent/sleeping, easily aroused with light tactile stimulation
3	Deeply sedated: deep sleep, arousable only with significant physical stimulation
4	Unarousable

**Table 2:** Comparison according to demographic characteristics.

Variables		Ketamine (*N* = 30)	Control (*N* = 30)	*P* value
Age (years), mean ± SD		28.7 ± 5.6	29.1 ± 7.5	^∧^0.815
Weight (kg), mean ± SD		83.5 ± 15.0	79.3 ± 13.8	^∧^0.264
BMI (kg/m^2^), mean ± SD		27.2 ± 2.2	26.3 ± 5.2	^∧^0.387
Gravidity (*n*, %)	Nulli	5 (16.7%)	8 (26.7%)	#0.347
	Multi	25 (83.3%)	22 (73.3%)	
ASA, (*n*, %)	II	30 (100.0%)	30 (100.0%)	—
Operation duration (minutes), mean ± SD		19.1 ± 4.0	18.0 ± 3.4	^∧^0.267

^∧^Independent *t*-test. #Chi square test.

**Table 3:** Comparison according to POMS (1/2).

Items	Time	Ketamine (*N* = 30)	Control (*N* = 30)	Effect of ketamine relative to control		RMANOVA *P* values		
				Mean ± SD	Between subjects	Between subjects	Within subjects	Inter-action
Vigor								
Active	Pre	1.0 ± 0.5	1.1 ± 0.5	−0.1 ± 0.1	−0.3–0.2	0.203	**<0.001** ^^*∗*^^	**0.004** ^^*∗*^^
	Post	2.2 ± 0.6	1.8 ± 0.6	0.4 ± 0.2	0.1–0.7			
Alert	Pre	1.4 ± 0.8	1.5 ± 0.9	−0.1 ± 0.2	−0.5–0.4	0.139	**0.005** ^^^*∗*^^^	**0.041** ^^*∗*^^
	Post	2.2 ± 1.0	1.6 ± 1.0	0.6 ± 0.3	0.1–1.1			
Energetic	Pre	0.8 ± 0.6	0.7 ± 0.7	0.2 ± 0.2	−0.2–0.5	**0.047** ^^*∗*^^	**<0.001** ^^*∗*^^	**0.010** ^^*∗*^^
	Post	2.2 ± 0.8	1.5 ± 1.2	0.7 ± 0.3	0.1–1.2			
Lively	Pre	0.3 ± 0.5	0.1 ± 0.4	0.1 ± 0.1	−0.1–0.4	0.300	**<0.001** ^^*∗*^^	0.999
	Post	0.7 ± 0.6	0.5 ± 0.7	0.1 ± 0.2	−0.2–0.5			
Total vigor subscale	Pre	3.5 ± 1.3	3.4 ± 1.8	0.2 ± 0.4	−0.6–1.0	**0.042** ^^*∗*^^	**<0.001** ^^*∗*^^	**0.004** ^^*∗*^^
	Post	7.2 ± 2.2	5.4 ± 2.8	1.8 ± 0.7	0.5–3.1			

Tension								
Anxious	Pre	1.7 ± 0.5	1.4 ± 1.0	0.3 ± 0.2	−0.1–0.7	**0.049** ^^*∗*^^	0.869	**<0.001** ^^*∗*^^
	Post	1.0 ± 0.7	2.0 ± 0.9	−1.0 ± 0.2	−1.4–0.6			
Nervous	Pre	1.3 ± 0.7	1.2 ± 0.9	0.1 ± 0.2	−0.3–0.5	**0.001** ^^*∗*^^	0.489	**<0.001** ^^*∗*^^
	Post	0.5 ± 0.6	1.8 ± 1.1	−1.3 ± 0.2	−1.8–0.9			
Panicky	Pre	1.2 ± 1.1	1.4 ± 0.9	−0.2 ± 0.3	−0.7–0.3	**<0.001** ^^*∗*^^	0.359	**<0.001** ^^*∗*^^
	Post	0.4 ± 0.5	2.0 ± 1.0	−1.6 ± 0.2	−2.0–1.2			
Worried	Pre	1.6 ± 0.8	1.6 ± 1.0	0.0 ± 0.2	−0.5–0.4	**<0.001** ^^*∗*^^	0.237	**<0.001** ^^*∗*^^
	Post	0.7 ± 0.6	2.2 ± 1.0	−1.5 ± 0.2	−1.9–1.0			
Total tension subscale	Pre	5.8 ± 1.9	5.6 ± 2.1	0.2 ± 0.5	−0.8–1.3	**<0.001** ^^*∗*^^	0.099	**<0.001** ^^*∗*^^
	Post	2.6 ± 1.5	8.0 ± 2.3	−5.4 ± 0.5	−6.4–4.4			

Fatigue								
Exhausted	Pre	1.3 ± 0.5	1.5 ± 1.2	−0.2 ± 0.2	−0.7–0.3	**<0.001** ^^*∗*^^	**<0.001** ^^*∗*^^	**<0.001** ^^*∗*^^
	Post	1.0 ± 0.4	2.6 ± 1.2	−1.7 ± 0.2	−2.1–1.2			
Sleepy	Pre	1.3 ± 0.7	1.2 ± 0.9	0.1 ± 0.2	−0.3–0.5	0.358	**<0.001** ^^*∗*^^	0.053
	Post	1.7 ± 0.6	2.1 ± 1.1	−0.4 ± 0.2	−0.9–0.1			
Tired	Pre	2.1 ± 0.8	1.9 ± 1.0	0.1 ± 0.2	−0.3–0.6	**<0.001** ^^*∗*^^	0.110	**<0.001** ^^*∗*^^
	Post	0.8 ± 0.5	2.8 ± 1.0	−2.0 ± 0.2	−2.4–1.5			
Worn out	Pre	1.0 ± 0.8	1.1 ± 0.9	−0.2 ± 0.2	−0.6–0.3	**<0.001** ^^*∗*^^	**<0.001** ^^*∗*^^	**<0.001** ^^*∗*^^
	Post	0.3 ± 0.5	2.5 ± 1.3	−2.2 ± 0.2	−2.7–1.7			
Total fatigue subscale	Pre	5.6 ± 1.8	5.8 ± 2.6	−0.2 ± 0.6	−1.3–1.0	**<0.001** ^^*∗*^^	**<0.001** ^^*∗*^^	**<0.001** ^^*∗*^^
	Post	3.7 ± 1.0	10.0 ± 3.8	−6.2 ± 0.7	−7.7–4.8			

^∧^RMANOVA test. ^^*∗*^^Significant.

**Table 4:** Comparison according to POMS (2/2).

Items	Time	Ketamine (*N* = 30)	Control (*N* = 30)	Effect of ketamine relative to control		RMANOVA *P* values		
				Mean ± SD	Between subjects	Between subjects	Within subjects	Inter-action
Depression								
Depressed	Pre	1.8 ± 0.8	1.8 ± 1.1	0.0 ± 0.2	−0.5–0.5	**0.002** ^*∗*^	0.355	**<0.001** ^*∗*^
	Post	1.0 ± 0.6	2.4 ± 1.2	−1.5 ± 0.2	−2.0–1.0			
Down hearted	Pre	1.8 ± 0.5	1.7 ± 1.1	0.1 ± 0.2	−0.4–0.6	**0.036** ^*∗*^	0.141	**<0.001** ^*∗*^
	Post	1.4 ± 0.6	2.3 ± 1.0	−0.9 ± 0.2	−1.3–0.5			
Miserable	Pre	1.8 ± 0.8	1.9 ± 1.1	−0.1 ± 0.2	−0.6–0.4	**0.002** ^*∗*^	0.261	**<0.001** ^*∗*^
	Post	1.3 ± 0.9	2.6 ± 1.0	−1.4 ± 0.2	−1.9–0.9			
Unhappy	Pre	2.8 ± 1.2	3.1 ± 1.1	−0.3 ± 0.3	−0.9–0.3	**0.005** ^*∗*^	**<0.001** ^*∗*^	**0.001** ^*∗*^
	Post	1.6 ± 0.8	2.8 ± 1.1	−1.1 ± 0.2	−1.6–0.7			
Total depression subscale	Pre	8.1 ± 2.3	8.4 ± 2.8	−0.3 ± 0.7	−1.6–1.1	**<0.001** ^*∗*^	**0.005** ^*∗*^	**<0.001** ^*∗*^
	Post	5.3 ± 1.9	10.2 ± 3.4	−4.9 ± 0.7	−6.3–3.4			

Confusion								
Confused	Pre	1.1 ± 0.9	1.1 ± 1.0	0.0 ± 0.2	−0.4–0.5	**0.005** ^*∗*^	0.430	**<0.001** ^*∗*^
	Post	0.5 ± 0.5	1.6 ± 0.7	−1.1 ± 0.2	−1.4–0.8			
Mixed up	Pre	1.3 ± 0.7	1.2 ± 1.0	0.1 ± 0.2	−0.4–0.5	0.177	**0.001** ^*∗*^	**<0.001** ^*∗*^
	Post	1.2 ± 0.8	1.9 ± 0.9	−0.6 ± 0.2	−1.1–0.2			
Muddled	Pre	1.7 ± 1.2	1.5 ± 0.8	0.2 ± 0.3	−0.3–0.7	**0.001** ^*∗*^	0.119	**0.001** ^*∗*^
	Post	0.6 ± 0.7	2.1 ± 1.0	−1.5 ± 0.2	−2.0–1.1			
Uncertain	Pre	1.3 ± 1.2	1.3 ± 0.9	0.1 ± 0.3	−0.5–0.6	**0.001** ^*∗*^	0.536	**<0.001** ^*∗*^
	Post	0.6 ± 0.7	2.2 ± 1.0	−1.6 ± 0.2	−2.0–1.2			
Total confusion subscale	Pre	5.4 ± 3.1	5.0 ± 2.1	0.4 ± 0.7	−1.0–1.7	**<0.001** ^*∗*^	0.603	**<0.001** ^*∗*^
	Post	2.9 ± 1.6	7.7 ± 2.1	−4.9 ± 0.5	−5.8–3.9			

Anger								
Angry	Pre	1.0 ± 0.7	1.1 ± 1.0	−0.2 ± 0.2	−0.6–0.3	**0.001** ^*∗*^	0.366	**<0.001** ^*∗*^
	Post	0.4 ± 0.5	1.5 ± 0.8	−1.1 ± 0.2	−1.5–0.7			
Annoyed	Pre	1.7 ± 0.6	1.5 ± 0.7	0.2 ± 0.2	−0.1–0.6	0.752	0.252	**0.007** ^*∗*^
	Post	1.5 ± 0.7	1.9 ± 0.9	−0.3 ± 0.2	−0.7–0.1			
Bad tempered	Pre	2.2 ± 0.8	2.1 ± 1.2	0.1 ± 0.3	−0.5–0.6	0.715	**<0.001** ^*∗*^	0.214
	Post	1.5 ± 0.7	1.7 ± 1.2	−0.2 ± 0.2	−0.7–0.3			
Bitter	Pre	1.9 ± 1.4	1.7 ± 1.3	0.1 ± 0.3	−0.5–0.8	0.552	**0.005** ^*∗*^	**0.010** ^*∗*^
	Post	1.2 ± 1.2	1.7 ± 1.3	−0.5 ± 0.3	−1.1–0.1			
Total anger subscale	Pre	6.7 ± 2.5	6.4 ± 1.9	0.3 ± 0.6	−0.9–1.4	0.095	**<0.001** ^*∗*^	**<0.001** ^*∗*^
	Post	4.7 ± 2.0	6.8 ± 2.6	−2.2 ± 0.6	−3.4–1.0			

Total POMS								
Preoperative		28.1 ± 8.0	27.8 ± 9.5	0.3 ± 2.3	−4.3–4.8	**<0.001** ^*∗*^	**<0.001** ^*∗*^	**<0.001** ^*∗*^
Postoperative		11.9 ± 5.4	37.3 ± 13.0	−25.4 ± 2.6	−30.5–20.2			

^∧^RMANOVA test. ^*∗*^Significant.

**Table 5:** Comparison according to postoperative sedation, side effects, and satisfaction.

Variables		Ketamine (*N* = 30)	Control (*N* = 30)	*P* value	Effect of ketamine relative to control	
					Mean ± SD	95% CI
Postoperative sedation						
Minute 0		1.3 ± 0.5	0.3 ± 0.5	**^∧^<0.001** ^*∗*^	1.0 ± 0.1	0.8–1.2
Minute 60		0.2 ± 0.4	0.0 ± 0.0	**^∧^0.023** ^*∗*^	0.2 ± 0.1	0.0–0.3
Minute 120		0.1 ± 0.3	0.0 ± 0.0	^∧^0.155	0.1 ± 0.0	0.0–0.2

Satisfaction						
Satisfaction		1.6 ± 0.5	2.8 ± 0.8	**^∧^<0.001** ^*∗*^	-1.2 ± 0.2	−1.5–0.8

Perioperative side effects						
					RR (95% CI)	
Headache	Intraoperative	6 (20.0%)	0 (0.0%)	**§0.024** ^*∗*^	—	
	Postoperative	7 (23.3%)	6 (20.0%)	#0.754	1.17 (0.44–3.07)	
Diplopia	Intraoperative	9 (30.0%)	0 (0.0%)	**§0.002** ^*∗*^	—	
	Postoperative	6 (20.0%)	0 (0.0%)	**§0.024** ^*∗*^	—	

Perioperative psychedelic phenomena						
Hallucination		2 (6.7%)	0 (0.0%)	§0.492	—	
Dissociation		6 (20.0%)	0 (0.0%)	**§0.024** ^*∗*^	—	
Relaxation		25 (83.3%)	0 (0.0%)	**#<0.001** ^*∗*^	—	
Euphoria		7 (23.3%)	0 (0.0%)	**§0.011** ^*∗*^	—	
Dysphoria		1 (3.3%)	0 (0.0%)	§0.999	—	

^∧^Independent *t*-test. #Chi square test. §Fisher's Exact test. ^*∗*^Significant. RR: relative risk. CI: confidence interval. ^*∗*^Significant.

## Data Availability

The datasets used and/or analyzed during the current study are available from the corresponding author on reasonable request.

## Additional Points

*Keypoints.* (i) Emotional and psychological effects after abortion are more common than physical effects. (ii) We studied the effect of ketamine on postoperative mood after D&C procedure. (iii) Ketamine infusion led to improved mood scores but it was not without side effects. (iv) If ketamine is used in the context of a protocol, it can be very effective and safe. (v) Further future studies exploring postoperative mood scores after 24 hours post-infusion are needed.

## Supplementary Materials

Supplementary Materials Ethical approval statement: Trial ID PACTR201907779292947. Consort Check list.
