# Supplementary material for: Acute Effects of Ketamine Infusion on Postoperative Mood Scores in Patients Undergoing Dilation and Curettage: A Randomized Double-Blind Controlled Study
Source: Anesthesiol Res Pract. 2021 Mar 23;2021:6674658. doi: 10.1155/2021/6674658 (PMC8009713; doi:10.1155/2021/6674658)
Supplement: Supplementary Materials — Ethical approval statement: Trial ID PACTR201907779292947. Consort Check list. [file 6674658.f1.zip › 6674658.f1/Trial ID PACTR201907779292947.pdf]

Pan African Clinical Trials Registry

South African Medical Research Council, South African Cochrane Centre
PO Box 19070, Tygerberg, 7505, South Africa
Telephone: +27 21 938 0506 / +27 21 938 0834 Fax: +27 21 938 0836
Email: pactradmin@mrc.ac.za Website: www.pactr.org

Trial #: PACTR201907779292947

Date of Approval:	16/07/2019	Trial Status:	Registered in accordance with WHO and ICMJE standards

TRIAL DESCRIPTION

**Public title**	Effect of ketamine infusion on postoperative mood scores in patients undergoing D&C. A randomized double blinded controlled study
**Official scientific title**	Effect of ketamine infusion on postoperative mood scores in patients undergoing D&C. A randomized double blinded controlled study
**Brief summary describing the background and objectives of the trial**	Emotional and psychological effects following abortion are more common than physical side effects and can range from mild regret to more serious complications such as depression. Research published in 2011 concluded that there was a "moderate to highly increased risk of mental health problems after abortion." The researchers suggested that undergoing a termination increased the risk by 81 percent and attributed 10 percent of this risk to the termination itself. Women with a higher probability of having a negative emotional or psychological side effect include: Individuals who obtain an abortion in the later stages of pregnancy, Individuals without support from significant others or their partner, Women obtaining an abortion for genetic or fetal abnormalities [1] The Profile of Mood States (POMS) is a psychological rating scale published in 1971 used to assess transient, distinct mood states. This scale was developed by McNair, Droppleman, and Lorr [2]. The first edition of the Profile of Mood States scale is known as the POMS-Standard version or the POMS-long form. Multiple short versions were published later after rigorous validation processes. Advantages of using this assessment include the simplicity of administration and ease of participant understanding. POMS test allowed both positive and negative states to be measured and observed in a clinical setting. Furthermore, it may be used to measure the effectiveness of treatments such as, various psychotropic drug treatments. [3] Recent studies suggest that a single low-dose administration of ketamine can provide within a few minutes of its initiation a long-lasting effect on mood [4]. Ketamine has emerged as a rapid powerful antidepressant and anxiolytic with enduring effects that last a week after a single subanaesthetic dose in the range of 0.3 to 0.5mg/kg however, its underlying mechanisms are not clear [5] Interestingly, the half-life of ketamine is only 3 h, suggesting that its antidepressant effect is unrelated to continuous blocking of N-methyl-d-aspartate (NMDA) receptors; instead, ketamine may mediate synaptic plasticity to cause long-term behavioral changes [6].
**Type of trial**	RCT
**Acronym (If the trial has an acronym then please provide)**	
**Disease(s) or condition(s) being studied**	Anaesthesia,Mental and Behavioural Disorders,Nervous System Diseases
**Sub-Disease(s) or condition(s) being studied**	
**Purpose of the trial**	Prevention
**Anticipated trial start date**	19/07/2019
**Actual trial start date**	20/07/2019
**Anticipated date of last follow up**	31/08/2020
**Actual Last follow-up date**	31/08/2020
**Anticipated target sample size (number of participants)**	60
**Actual target sample size (number of participants)**	60
**Recruitment status**	Completed
**Publication URL**	

Secondary Ids	Issuing authority/Trial register

STUDY DESIGN

Intervention assignment	Allocation to intervention	If randomised, describe how the allocation sequence was generated	Describe how the allocation sequence/code was concealed from the person allocating the participants to the intervention arms	Masking	If masking / blinding was used
Parallel: different	Randomised	Simple randomization	Sealed opaque envelopes	Masking/blinding used	Care giver/Provider,Outcome

groups receive different interventions at same time during study		using a randomization table created by a computer software program			Assessors,Participants

INTERVENTIONS

Intervention type	Intervention name	Dose	Duration	Intervention description	Group size	Nature of control
Control Group	Control Group	Group C: patients received 50 ml normal saline over a period of 20 minutes.	20 minutes	The women were randomized to 2 groups: C (Control group) and K (Ketamine group) using random number tables. The assigned treatments were written on cards and sealed in secure opaque envelopes numbered in sequence. On the scheduled time of operation, the head nurse (who was not part of the study) opened each envelope just before surgery, prepared the infusion solution and handled it to the anesthesiologist who was blinded to the solution. He was then determined for collecting perioperative data. Group C: patients received 50 ml normal saline over a period of 20 minutes.	30	Placebo
Experimental Group	Ketamine Group	Group K: patients received 0.4 mg/kg ketamine diluted in 50 ml normal saline over 20 min	20 minutes	The women were randomized to 2 groups: C (Control group) and K (Ketamine group) using random number tables. The assigned treatments were written on cards and sealed in secure opaque envelopes numbered in sequence. On the scheduled time of operation, the head nurse (who was not part of the study) opened each envelope just before surgery, prepared the infusion solution and handled it to the anesthesiologist who was blinded to the solution. He was then determined for collecting perioperative data. Group K: patients received 0.4 mg/kg ketamine diluted in 50 ml normal saline over 20 min	30	

ELIGIBILITY CRITERIA

List inclusion criteria	List exclusion criteria	Age Category	Minimum age	Maximum age	Gender
18 years or older, physical status ASA I&II, with up to 12-week gestation, submitted to manual intrauterine aspiration for incomplete or retained abortion, participated in this randomized, double blind study.	patients with infected abortion or with psychiatric disorder on chronic medical treatment. Also, patients with pulmonary, hepatorenal, neuromuscular diseases body mass index over 30 kg/m2, usage of sedative drugs or substance abuse, any contraindications to regional anesthesia and finally emergency curettage for massive bleeding or hemodynamic instability	Adolescent: 13 Year-18 Year,Adult: 19 Year-44 Year,Middle Aged: 45 Year(s)-64 Year(s)	18 Year(s)	60 Year(s)	Female

ETHICS APPROVAL

Has the study received appropriate ethics committee approval	Date the study will be submitted for approval	Date of approval	Name of the ethics committee
Yes		10/07/2019	Research Ethics Committee REC

Ethics Committee Address

Street address	City	Postal code	Country
Abbasia	Cairo	11591	Egypt

OUTCOMES

Type of outcome	Outcome	Timepoint(s) at which outcome measured
Primary Outcome	Our primary outcome is mood changes which were assessed preoperatively and 2 h postoperatively with POMS-A to determine if there were differences in mood state that could be attributed to the interventional drug infused.	preoperatively and 2 h postoperatively
Secondary Outcome	Secondary outcomes were: 1. Mean blood pressure and heart rate were measured preoperatively then every 5 min till end of the operation. Postoperatively, mean blood pressure, heart rate, were recorded every 30 minutes for 2 hours in PACU 2. Total dose of ketamine infused. 3. The duration of surgery in minutes. 4. Presence of psychedelic' phenomenon [10] intraoperatively (during infusion) and/or postoperatively. It is a temporary altered state of consciousness that might be induced by ketamine infusion. The subjective phenomenon that might be reported included one or more of the following: perceptual disorders, a sense of detachment from their bodies	preoperatively and 2 h postoperatively

	(dissociation), a sense of relaxation or well-being. Hallucinations, if occurred would be treated with injecting 2 mg intravenous midazolam. 5. Presence of intraoperative (during infusion) and/or postoperative other side effects as headache & diplopia 6. Presence of intraoperative (during infusion) and/or postoperative nausea and vomiting (PONV) which was rated on a 4-point scale (0=no PONV, 1=Mild nausea, 2=Severe nausea, 3=Vomiting). If PONV scale was 2 or more, ondansetron 4mg was given intravenously. 7. The patient satisfaction was assessed postoperatively at the 2nd hour. Patient satisfaction were rated on a scale of 1 to 4 (1=perfect, 2=good, 3=moderate, 4=bad). 8. Assessment of postoperative pain was done with the aid of visual analogue scale (VAS) in which patients were requested to estimate their pain on vertical VAS 0–10 cm where (0) is marked as no pain and (10) is marked as the worst pain ever felt. [11]. This was recorded postoperatively at specific timings: every 30 minutes for 2 hours postoperatively. 30 mg ketorolac was given intravenously if VAS score was > or equal to 4 at any of the mentioned times (with a maximum total ketorolac dose of 60 mg). 9. Assessment of perioperative sedation level using Sedation scale using University of Michigan Sedation Scale (UMSS) [12] The UMSS as a measure of sedation during procedures. The UMSS is a simple observational tool that assesses the level of alertness on a five-point scale ranging from 1 (wide awake) to 5 (unarousable with deep stimulation). Table (1) It will be assessed intraopertively till the 2nd postoperative hour; every 30 minutes.	

RECRUITMENT CENTRES

Name of recruitment centre	Street address	City	Postal code	Country
Ain Shams University hospital	Abbaseya	Cairo		Egypt

FUNDING SOURCES

Name of source	Street address	City	Postal code	Country
Ashraf Nabil Raham Hasan Mohamed Abdulmohsen Abdulnaiem Ismaiel	Abbasiya	Cairo		Egypt

SPONSORS

Sponsor level	Name	Street address	City	Postal code	Country	Nature of sponsor
Primary Sponsor	Ain Shams University	Abbasiya	Cairo		Egypt	Hospital

COLLABORATORS

Name	Street address	City	Postal code	Country
Ashraf Nabil Saleh	5th Settlement	Cairo		Egypt
Raham Hasan Mostafa	Nasr City	Cairo		Egypt
Mohamed Abdulmohsen Abdulnaiem Ismaiel	Heliopolis	Cairo		Egypt

CONTACT PEOPLE

Role	Name	Email	Phone	Street address
**Principal Investigator**	Raham Hasan Mostafa	rahamhasan@yahoo.com	01222530020	Nasr City
**City**	**Postal code**	**Country**	**Position/Affiliation**	
Cairo		Egypt	Assistant professor Ain Shams University	
**Role**	**Name**	**Email**	**Phone**	**Street address**
**Public Enquiries**	Mohamed Abdulmohsen Abdulnaiem Ismaiel	mohamedismaiel79@gmail.com	01001381193	Heliopoless
**City**	**Postal code**	**Country**	**Position/Affiliation**	
Cairo		Egypt	Lecturer of Anesthesia Ain Shams University	
**Role**	**Name**	**Email**	**Phone**	**Street address**
**Scientific Enquiries**	Ashraf Nabil Saleh	ashraf_nabel@med.asu.edu.eg	01001100613	5th Settlement
**City**	**Postal code**	**Country**	**Position/Affiliation**	
Cairo		Egypt	Lecturer of Anesthesia Ain Shams University	

REPORTING

Share IPD	Description	Additional Document Types	Sharing Time Frame	Key Access Criteria
Yes	Excel Sheet	Statistical Analysis Plan	Twelve months	Only journal editors to whom manuscript will be applied to
**URL**	**Results Available**	**Results Summary**	**Result Posting Date**	**First Journal Publication Date**

Not Available	No			
**Result Upload 1:**	**Result Upload 2:**	**Result Upload 3:**	**Result Upload 4:**	**Result Upload 5:**
**Results URL Hyperlinks**	**Link To Protocol**			
Result URL Hyperlinks				

Changes to trial information

Section Name	Field Name	Date	Reason	Old Value	Updated Value
Eligibility	Age group	16/07/2019	Correction to include minimum age of 18 years	Adult: 19 Year-44 Year, Middle Aged: 45 Year(s)-64 Year(s)	Adolescent: 13 Year-18 Year, Adult: 19 Year-44 Year, Middle Aged: 45 Year(s)-64 Year(s)
**Section Name**	**Field Name**	**Date**	**Reason**	**Old Value**	**Updated Value**
Funding Source	FundingSources List	16/07/2019	As requestd by reviewers		Ashraf Nabil Raham Hasan Mohamed Abdulmohsen Abdulnaiem Ismaiel, Abbasiya, Cairo, , Egypt, Self Funded,
**Section Name**	**Field Name**	**Date**	**Reason**	**Old Value**	**Updated Value**
Collaborators	Collaborators List	16/07/2019	As requested by reviewers		Ashraf Nabil Saleh, 5th Settlement, Cairo, , Egypt
**Section Name**	**Field Name**	**Date**	**Reason**	**Old Value**	**Updated Value**
Collaborators	Collaborators List	16/07/2019	As requested by reviewers		Raham Hasan Mostafa, Nasr City, Cairo, , Egypt
**Section Name**	**Field Name**	**Date**	**Reason**	**Old Value**	**Updated Value**
Collaborators	Collaborators List	16/07/2019	As requested by reviewers		Mohamed Abdulmohsen Abdulnaiem Ismaiel, Heliopolis, Cairo, , Egypt
**Section Name**	**Field Name**	**Date**	**Reason**	**Old Value**	**Updated Value**
Reporting	Plan to share IPD	16/07/2019	As requested by reviewers	No	Yes
**Section Name**	**Field Name**	**Date**	**Reason**	**Old Value**	**Updated Value**
Reporting	IPD description	16/07/2019	As requested by reviewers		Excel Sheet
**Section Name**	**Field Name**	**Date**	**Reason**	**Old Value**	**Updated Value**
Reporting	IPD-Sharing time frame	16/07/2019	As requested by reviewers		Twelve months
**Section Name**	**Field Name**	**Date**	**Reason**	**Old Value**	**Updated Value**
Reporting	Key access criteria	16/07/2019	As requested by reviewers		Only journal editors to whom manuscript will be applied to
**Section Name**	**Field Name**	**Date**	**Reason**	**Old Value**	**Updated Value**
Reporting	IPD URL	16/07/2019	As requested by reviewers		Not Available
**Section Name**	**Field Name**	**Date**	**Reason**	**Old Value**	**Updated Value**
Reporting	Study protocol document	16/07/2019	As requested by reviewers		Statistical Analysis Plan
**Section Name**	**Field Name**	**Date**	**Reason**	**Old Value**	**Updated Value**
Trial Information	Anticipated date of last follow up	31/08/2020	Study completed	30 Sep 2019	31 Aug 2020
**Section Name**	**Field Name**	**Date**	**Reason**	**Old Value**	**Updated Value**
Trial Information	Completion date	31/08/2020	Study completed	30 Sep 2019	31 Aug 2020
**Section Name**	**Field Name**	**Date**	**Reason**	**Old Value**	**Updated Value**
Trial Information	Recruitment status	31/08/2020	Study completed	Not yet recruiting	Completed
